# New aspects in the management of pneumonia

**DOI:** 10.1186/s13054-016-1442-y

**Published:** 2016-10-01

**Authors:** Elena Prina, Adrian Ceccato, Antoni Torres

**Affiliations:** 1 Servei de Pneumologia, Institut del Torax, Hospital Clinic, IDIBAPS, Universitat de Barcelona, Barcelona, Spain; 2 Centro de Investigación Biomedica En Red-Enfermedades Respiratorias (CibeRes, CB06/06/0028), Barcelona, Spain; 3 Seccion Neumologia, Hospital Nacional Alejandro Posadas, Palomar, Argentina; 4 UVIR, Servei de Pneumologia, Hospital Clínic, Villarroel 170., 08036 Barcelona, Spain

**Keywords:** Community-acquired pneumonia, Corticosteroid, Immunoglobulin

## Abstract

Despite improvements in the management of community-acquired pneumonia (CAP), morbidity and mortality are still high, especially in patients with more severe disease. Early and appropriate antibiotics remain the cornerstone in the treatment of CAP. However, two aspects seem to contribute to a worse outcome: an uncontrolled inflammatory reaction and an inadequate immune response. Adjuvant treatments, such as corticosteroids and intravenous immunoglobulins, have been proposed to counterbalance these effects. The use of corticosteroids in patients with severe CAP and a strong inflammatory reaction can reduce the time to clinical stability, the risk of treatment failure, and the risk of progression to acute respiratory distress syndrome. The administration of intravenous immunoglobulins seems to reinforce the immune response to the infection in particular in patients with inadequate levels of antibodies and when an enriched IgM preparation has been used; however, more studies are needed to determinate their impact on outcome and to define the population that will receive more benefit.

## Background

Despite the use of early and appropriate antibiotic treatment, mortality related to community-acquired pneumonia (CAP) is still high [1], especially in patients with severe disease. Previous studies have shown that approximately 18 % of patients hospitalized for CAP matched the criteria for severe CAP. These patients more frequently present with septic shock and need for mechanical ventilation, with a mortality of approximately 29 % [2]. In addition to the infection, septic shock is generally thought to be caused by an excessive or uncontrolled pro-inflammatory response [3].

Pneumonia is a complex disease caused by the action of pathogens and the local and systemic inflammatory responses of the patient. A stronger inflammatory response has been shown to be associated with treatment failure and mortality [4]. In particular, high levels of interleukin (IL)-6, IL-8, and IL-10 have been detected in patients with severe pneumonia and excess IL-6 and IL-10 was associated with increased mortality (from 4.8 to 11.4 %) [5, 6].

Moreover, in some patients with CAP, excessive levels of cytokines can be released (called the Jarisch–Herxheimer-like reaction) after the initiation of antibiotics, causing damage similar to other infections characterized by high bacterial load (e.g., meningococcal meningitis) [7, 8].

Another aspect regarding the immune response to the infection is that low levels of immunoglobulins are found, particularly in patients with recurrent episodes of pneumonia, and may be responsible for the predisposition to recurrent infections and worse outcome [9].

Considering that pathogens resistant to the empiric antibiotic treatment are not a common cause of CAP, two aspects seem to contribute to a worse outcome: an uncontrolled inflammatory reaction and an inadequate immune response. Adjuvant treatments, such as corticosteroids and intravenous immunoglobulins, have been proposed to counterbalance these effects.

## Corticosteroids in CAP

### Corticosteroids: mechanism of action

During an infection, endogenous corticosteroids are produced by the activation of the hypothalamic–pituitary–adrenal axis with the aim of controlling excessive inflammation. The free cortisol, which is the active form of the hormone, induces the expression of anti-inflammatory proteins and inhibition of proinflammatory proteins [10].

Glucocorticosteroid drugs reproduce effects similar to endogenous cortisol: they have anti-inflammatory activity by switching genes on and off, resulting in a reduction of inflammatory cytokines and chemokines. Corticosteroids have an effect on structural cells of the respiratory tract: they act on epithelial cells by inhibiting transcription factors such as NF-kB, on mucous glands by decreasing mucus secretion, and on smooth muscle cells by increasing β2 receptors [11].

Another aspect that may contribute to the beneficial effect of corticosteroid treatment is related to the presence of adrenal insufficiency or inadequate adrenal function in some cases of severe CAP [12].

In an animal model of mechanically ventilated piglets with pneumonia due to *Pseudomonas aeruginosa*, we demonstrated the presence of lower bacterial burden in the lungs and less severe histological pneumonia in the group treated with antibiotics plus corticosteroids in comparison with the group treated with antibiotics plus placebo, suggesting a potential beneficial effect of corticosteroids on bacterial burden and lung tissue severity, in addition to the systemic inflammatory response [13]. In acute respiratory distress syndrome (ARDS), the presence of high levels of cytokines is associated with a higher risk of nosocomial infection because the inflammatory biomarkers appear to favor bacterial growth. Meduri et al. [14], in an in vitro study, showed that the addition of methylprednisone to monocytes stimulated by lipopolysaccharide can increase the ability to suppress bacterial replication.

### Studies evaluating the effect of corticosteroids in CAP

The main studies on corticosteroids in pneumonia are summarized in Table 1 [15–26]. Several studies have evaluated the effects of corticosteroids in CAP. The first studies and meta-analyses included a heterogeneous population evaluating different outcomes, resulting in controversial data.

**Table 1:** Studies on corticosteroids in CAP

Reference	Study design and population	Main results
Confalonieri et al. 2005 [15]	Multicenter RCT	Improvement in PaO_2_/FiO_2_ (*p* = 0.002), chest radiograph score (*p* < 0.0001), reduction in C-reactive protein levels (*p* = 0.01), delayed septic shock (*p* = 0.001), reduction in length of hospital stay (*p* = 0.03), and mortality (*p* = 0.009)
	Hydrocortisone versus placebo	
	Patients with severe CAP	
Garcia-Vidal et al. 2007 [16]	Retrospective observational study	Systemic steroids were independently associated with decreased mortality (OR 0.287; 95 % CI 0.113–0.732).
	Patients with severe CAP	
Snijders et al. 2010 [17]	Unicentre RCT in Netherlands	Clinical cure at day 7 was 80.8 % in the prednisolone group and 85.3 % in the placebo group (*p* = 0.38)
	Prednisolone versus placebo	Clinical cure at day 30 was 66.3 % in the prednisolone group and 77.1 % in the placebo group (*p* = 0.08).
	Hospitalized patients with CAP	Late failure (>72 h after admission) was more common in the prednisolone group than in the placebo group (19.2 versus 6.4 %, respectively; *p* = 0.04).
Meijvis et al. 2011 [18]	Bicenter RCT in Netherlands	Reduction in length of stay in dexamethasone group compared with the placebo group (6.5 versus 7.5 days, respectively; *p* = 0.048)
	Dexamethasone versus placebo	
	Patients with CAP	
Chen et al. 2011 [19]	Meta-analysis	Accelerated the resolution of symptoms or time to clinical stability and decreased the rate of relapse of the disease
	Patients with pneumonia	
Nie et al. 2012 [20]	Meta-analysis	Corticosteroids did not significantly reduce mortality in the general population (Peto OR = 0.62, 95 % CI 0.37–1.04; *p* = 0.07). A survival benefit was found in a subgroup of patients with severe CAP (Peto OR = 0.26, 95 % CI 0.11–0.64; *p* = 0.003).
	Patients with CAP	
Shafiq et al. 2013 [21]	Meta-analysis	Reduced hospital length of stay with the use of corticosteroids (mean −1.21 days, 95 % CI –2.12 to −0.29)
	Patients with CAP	No effect on hospital mortality
Cheng et al. 2014 [22]	Meta-analysis	Use of corticosteroids significantly reduced hospital mortality compared with placebo (Peto OR = 0.39, 95 % CI 0.17–0.90)
	Patients with severe CAP	
Torres et al. 2015 [23]	Multicenter RCT in Spain	Corticosteroid treatment reduced the risk of treatment failure (OR = 0.34, 95 % CI 0.14–0.87; *p* = 0.02)
	Methylprednisolone versus placebo	In-hospital mortality did not differ between the two groups (10 % in the methylprednisolone group versus 15 % in the placebo group; *p* = 0.37)
	Patients with severe CAP and high inflammatory response	
Blum et al. 2015 [24]	Multicenter RCT in Switzerland	Reduction of time to clinical stability in the prednisone group compared with the placebo group (3 days versus 4.4 days, respectively; *p* < 0.0001)
	Prednisolone versus placebo	
	Patients with CAP	
Siemieniuk et al. 2015 [25]	Meta-analysis	Corticosteroids were associated with possible reductions in all-cause mortality (RR 0.67, 95 % CI 0.45–1.01), need for mechanical ventilation (RR 0.45, 95 % CI 0.26–0.79], and ARDS (RR 0.24, 95 % CI 0.10–0.56]). Corticosteroids decreased time to clinical stability (mean difference −1.22 days, 95 % CI −2.08 to −0.35 days), and duration of hospitalization (mean difference −1.00 day, 95 % CI −1.79 to −0.21 days)
	Patients with CAP	
Wan et al. 2016 [26]	Meta-analysis	Corticosteroids did not have an effect on mortality (RR 0.72, 95 % CI 0.43–1.21) in patients with CAP and patients with severe CAP (RCTs: RR 0.72, 95 % CI 0.43–1.21). Corticosteroid treatment was associated with a decreased risk of ARDS (RR 0.21, 95 % CI 0.08–0.59)
	Patients with CAP	

*ARDS* acute respiratory distress syndrome, *CAP* community acquired pneumonia, *CI* confidence interval, *OR* odds ratio, *RCT* randomized controlled trial, *RR* relative risk

A Cochrane meta-analysis [19] selected six randomized controlled trails (RCTs) of corticosteroids in pneumonia including a total of 437 participants. The use of corticosteroids accelerated the resolution of symptoms and time to clinical stability (defined as improvement in chest X-ray and normalization of temperature, respiratory rate, and inflammatory markers). Corticosteroids provided no benefit in terms of mortality and the authors concluded that it was not possible to make any definitive recommendations because the studies analyzed in the meta-analysis did not provide strong evidence. Another meta-analysis [20] including nine RCTs with a total of 1001 patients showed that the use of corticosteroids was not associated with significantly lower mortality considering all the patients (odds ratio (OR) 0.62, 95 % confidence interval (CI) 0.37–1.04; *p* = 0.07). However, a survival benefit was detected in the subgroup of patients with severe CAP (OR 0.26, 95 % CI 0.11–0.64; *p* = 0.003) and among patients receiving more prolonged corticosteroid treatment (OR 0.51, 95 % CI 0.26–0.97; *p* = 0.04). Prolonged corticosteroid treatment was defined as more than 5 days of corticosteroid treatment and a maximum of 9 days. In terms of adverse effects, corticosteroids increased the risk of hyperglycemia (OR 2.64, 95 % CI 1.68–4.15; *p* < 0.001) but did not increase the risk of superinfection (OR 1.36, 95 % CI 0.65–2.84; *p* = 0.41) or gastroduodenal bleeding (OR 1.67, 95 % CI 0.41– 6.80; *p* = 0.47).

In conclusion, these studies were not able to provide definitive results regarding the use of corticosteroids in CAP. The main limitations regarded the inclusion of a heterogeneous population in terms of severity (from mild to severe) and level of inflammatory response (e.g., defined by the level of C-reactive protein (CRP)) and the use, in some cases, of inadequate dosage of corticosteroids (low or excessively high).

### Randomized controlled trials

Two recent multicenter RCTs have been published regarding the use of corticosteroids in CAP.

In a multicenter, double-blind, randomized, placebo-controlled trial [24], a total of 785 patients with CAP were randomized to receive oral corticosteroids (50 mg of prednisone for 7 days) or placebo as adjunctive treatment. The corticosteroid group reported a shorter time to reach clinical stability in comparison with the placebo group (3 days versus 4.4 days). In the study, the time to clinical stability was defined as the days until reaching stable vital signs for 24 h or longer (including normalization of temperature, heart rate, spontaneous respiratory rate, systolic blood pressure, mental status, ability for oral intake, and adequate oxygenation on room air). The complications related to pneumonia were not significantly different in the groups whereas the prednisone group more frequently presented hyperglycemia needing insulin treatment (19 versus 11 %; OR 1.96; 95 % CI 1.31–2.93; *p* = 0.001). However, other adverse events typically associated with corticosteroid use (such as gastrointestinal bleeding, nosocomial infections) were rare and similar in both groups. The mortality rate, considered as a secondary outcome in the study, was not different in the two groups (n = 16 (4 %) in the prednisone group versus n = 13 (3 %) in the placebo group; *p* = 0.57). This study presented some limitations, in particular the use of a weak outcome such as clinical stability, which included some items such as temperature that could be influenced by the use of corticosteroids. Moreover, the majority of patients had a mild disease presentation, thereby decreasing result validation for the most severe diseases.

We recently published a multicentre RCT [23] where we compared patients with severe CAP and strong inflammatory response (defined by a CRP >150 mg/L) treated with corticosteroids plus antibiotics versus placebo plus antibiotics. We used intravenous methylprednisolone at a dose of 0.5/mg/kg every 12 h for 5 days. We included only patients with severe CAP, defined according to the modified American Thoracic Society criteria or by a Pneumonia Severity Index risk class V [27]. The patients receiving corticosteroids had significantly lower treatment failure in comparison with the placebo group (13 versus 31 %, respectively; *p* = 0.02). This difference was due to late treatment failure (developing between 72 and 120 h after treatment initiation) and, in particular, patients in the corticosteroid group showed a more evident effect on the reduction of radiological progression (2 versus 15 %; *p* = 0.007). Indeed, the use of corticosteroids reduced the risk of treatment failure by 18 % (95 % CI 3–32 %) in the intention to treat analysis. The association between treatment failure, with radiographic progression as a criterion, and mortality has been shown by Menendez et al. [5]. The protective effect of corticosteroids on radiographic progression could be interpreted as an effect preventing the development of ARDS or blocking the Jarisch–Herxheimer-like reaction [8]. No significant difference was observed in mortality between the two groups (10 % in the methylprednisolone arm versus 15 % in the placebo arm; *p* = 0.37); however, the study was not powered for mortality as this was not the primary outcome. Importantly, we detected no significant side effects in patients receiving corticosteroids. The strength of this study is the homogeneous population with severe CAP and a strong inflammatory response and the use of an outcome (treatment failure) closely associated with mortality [5]. The limitation of this study was the prolonged recruitment period.

**Fig. 1:**
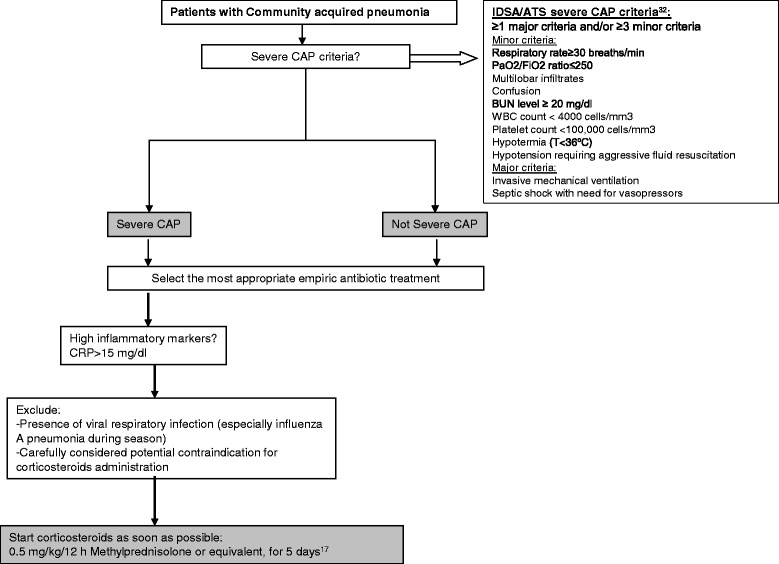
Flowchart for the management of patients with CAP

In Fig. 1 we propose a flowchart for the management of patients with severe CAP.

### Updated meta-analyses

A recent meta-analysis [26] including nine RCTs (1667 patients) and six cohort studies (4095 patients) showed that the use of corticosteroids is not associated with a significant reduction in mortality in patients with CAP (risk ratio (RR) 0.72; 95 % CI 0.43–1.21; evidence rank low) or in the subgroup of patients with severe CAP (RCT RR 0.72, 95 % CI 0.43–1.21, evidence rank low; cohort study RR 1.00, 95 % CI 0.86–1.17). However, corticosteroids produced a benefit in terms of reduction of ARDS (RR 0.21, 95 % CI 0.08–0.59), length of hospital and ICU stay, duration of IV antibiotics, and time to clinical stability without a significant increase in side effects.

In contrast, another meta-analysis [25] demonstrated a reduction in all causes of mortality in patients receiving corticosteroids (12 trials, 1974 patients, RR 0.67 [95 % CI 0.45–1.01], risk difference [RD] 2.8 %, moderate certainty). Moreover, the analysis confirmed the reduced risk of ARDS (RR 0.24 [95 % CI 0.10–0.56]), the need for mechanical ventilation, the decreased time to clinical stability and length of hospital stay, and increased episodes of hyperglycemia requiring treatment but no increase in the frequency of gastrointestinal hemorrhage.

In conclusion, all these studies confirm that the use of corticosteroids in CAP is associated with the following benefits: reduced length of hospital stay, reduced time to clinical stability, and prevention of ARDS.

No definitive answer is available yet regarding the effect of corticosteroids on the reduction of death and larger studies are needed to define the effect on mortality. In particular, some meta-analyses suggested that corticosteroids may decrease mortality in the subgroup of patients with severe CAP; however, these data have not been confirmed in other studies.

### Corticosteroids in pneumonia caused by influenza or less frequent pathogens

The effects of corticosteroids in some specific infections are the subject of debate. For example, a meta-analysis showed a benefit in *Pneumocystic jiroveci* pneumonia [28], although this result came from small RCTs.

In patients with CAP due to viral infection, the effects of corticosteroids are still not clear. In H1N1 infection, corticosteroid use was associated with a higher incidence of pneumonia and mortality. In a Chinese descriptive study of influenza A (H7N9) viral pneumonia, the subgroup of patients receiving very high doses of corticosteroids (>150 mg/d methylprednisolone or equivalent) had increased mortality but no significantly worse outcome was detected with low to moderate doses of corticosteroids (25–150 mg/d methylprednisolone or equivalent) [29].

A recent meta-analysis by Cochrane [30] of corticosteroids as adjunctive treatment in influenza found insufficient evidence to determine the efficacy of corticosteroids in these patients. Delaney et al. [31] recently published an observational multicenter study of patients with influenza A (H1N1pdm09)-related critical illness. The crude hospital mortality was higher in patients who received corticosteroids compared with patients without corticosteroid treatment (25.5 versus 16.4 %, *p* = 0.007). Nevertheless, after adjusting for potential confounders, the authors did not find a significant association between corticosteroids and mortality in this population.

It appears that the use of corticosteroids was associated with a higher mortality but this result has to be carefully interpreted because only observational studies of low quality were included and RTCs were not identified for the analysis. More studies are needed to clarify this point.

### Side effects of corticosteroids

**Fig. 2:**
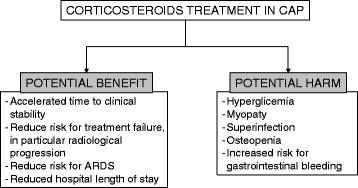
Potential harm and benefit of corticosteroids used in CAP

The main side effects associated with corticosteroids, especially with prolonged use, are hyperglycemia, myopathy, weight gain, brushing, and osteopenia (Fig. 2) [32]. As well as these side effects, corticosteroids have strong immunosuppressive effects, raising concerns regarding their use in acute infections, despite their potential effect in controlling excessive inflammatory response. The immunosuppressant effect of corticosteroids is related to dose and treatment duration. For example, the use of 40 mg of prednisolone per day for more than 1 week or 20 mg prednisolone or equivalent per day for a month can produce immunosuppression. In acute infection, a low dose for a short period (some days) may be useful for reducing inflammation and may not cause so much harm by producing immunosuppression. Moreover, a short period of corticosteroid treatment may reduce the risk for side effects.

Hyperglycemia is a frequent effect associated with corticosteroid use. This occurs in about 50 % of hospitalized patients receiving high doses of corticosteroids [33] and patients with chronic obstructive pulmonary disease (COPD) receiving oral corticosteroid treatment presented a more than fivefold risk of developing hyperglycemia. Hyperglycemia is associated with higher mortality and, in particular, hyperglycemia secondary to corticosteroids increased the risk of death by 10 % for each 18 mg/dL increase in blood glucose after adjusting for age, sex, and diabetes mellitus [34]. Detection of hyperglycemia in the first 24 h in patients with COPD reactivation was associated with worse outcomes [35]. On the other hand, strict management of glucose levels decreased morbidity and mortality in critically ill patients admitted to the surgical ICU [36]. For patients in the medical ICU, a benefit was shown in terms of morbidity but not mortality [37].

Myopathy is another side effect associated with acute and chronic corticosteroid treatment, especially in older patients, patients with cancer and respiratory muscle diseases, and physically inactive patients [38]. Corticosteroids induce myopathy by decreasing protein synthesis and increasing protein breakdown. This mechanism leads to muscle atrophy with a reduction of myofibrillar protein content and cross-sectional fiber area. In critically ill patients, the development of myopathy produces peripheral muscle weakness with longer duration of mechanical ventilation and an increased risk of nosocomial infections [39, 40].

### Corticosteroids and macrolides

There is a gap in knowledge regarding whether the beneficial anti-inflammatory effect of corticosteroids may be potentiated by the administration of a macrolide, which has an immunomodulatory effect.

The best antibiotic strategy for the treatment of CAP is currently a subject of debate. For severe CAP, international guidelines suggest the use of two antibiotics such as a β-lactam plus a macrolide or a β-lactam plus respiratory quinolone (levofloxacin or moxifloxacin) [40]. However, many observational studies and a recent meta-analysis have shown that the use of a β-lactam plus a macrolide is the best choice because it is associated with a better outcome and lower mortality in patients with severe CAP, especially in bacteremic pneumococcal CAP. The mechanisms responsible for the favorable effects related to the use of macrolides are not clear and have been attributed in part to their immunomodulatory effect, as observed in some studies [41]. In vitro and in vivo experimental models have shown that macrolides inhibit cytokine secretion by inflammatory and structural cells of the respiratory tract [42].

In patients admitted to the ward with non-severe CAP [43], monotherapy with a β-lactam was not inferior to a β-lactam plus a macrolide or fluoroquinolone in terms of 90-day mortality.

In another trial, the authors found no non-inferiority of β-lactam monotherapy in comparison with a β-lactam plus a macrolide in patients with moderate-severe CAP, considering as outcome the proportion of patients who did not reach clinical stability in 7 days [44].

In an experimental mouse model of *Mycoplasma pneumoniae* respiratory infection, the use of clarithromycin and dexamethasone was more effective than clarithromycin alone in decreasing levels of cytokines and histological signs of lung inflammation [45]. Another study in patients with non-responding pneumonia demonstrated a reduction in inflammatory biomarkers such as IL-6 and IL-8 in bronchoalveolar lavage in patients receiving treatment with corticosteroids plus a macrolide [46].

The combination of a macrolide plus a corticosteroid is currently used without scientific evaluation, although we do not know whether this combination may decrease the inflammatory response to a very low level, thereby increasing the risk of side effects. We therefore need to better investigate the effects of corticosteroids and macrolides together in order to provide data that may be used to support clinical indications for this combination in severe CAP.

### Corticosteroids in patients with COPD and pneumonia

Corticosteroids have proven benefits in the treatment of acute exacerbation of COPD and the presence of chronic respiratory disease is the main reason for adding corticosteroids to antimicrobial treatment in pneumonia [47].

Patients with COPD and CAP presented a different early inflammatory pattern compared with patients with CAP only. In particular, on the day of admission to hospital, the patients with COPD had lower levels of tumor necrosis factor (TNF), IL-1, and IL-6 but no differences in levels of CRP, procalcitonin, IL-8, and IL-10. These differences were mediated in part by corticosteroids; in fact, lower levels of TNF-α persisted after excluding patients who received inhaled and oral corticosteroids at home [48]. In contrast with this result, another study showed that COPD patients with CAP who had received prior treatment with inhaled corticosteroids had lower levels of TNF-α after adjusting for other confounders in comparison with the overall population [49].

In addition, another study found that on days 1 and 3, patients with CAP and a history of COPD had significantly higher levels of CRP, procalcitonin, TNF-α, and IL-6 than patients admitted with acute exacerbation of COPD [50]. These results were maintained after adjusting for inhaled pharmacotherapy.

In conclusion, patients with CAP and a history of COPD represent a specific population with a different inflammatory pattern and further studies are needed to clarify the use of corticosteroids in these patients during CAP episodes, especially in those receiving inhaled corticosteroids.

### Immunomodulatory effects of quinolones

Fluoroquinolones have also shown an immunomodulatory effect [51].

In vitro, fluoroquinolones favor the synthesis of IL-2 but reduces the production of IL-1 and TNF.

In vivo, they affect cellular and humoral immunity by attenuating cytokine responses. In addition, certain fluoroquinolones enhance hematopoiesis by increasing concentrations of colony-stimulating factors (CSFs) in the lung and in the bone marrow. CSFs have a role in the response to infections. In fact, CSF knockout mice developed lung infections and the administration of CSF in neutropenic mice with candida reduced the risk of mortality and lung injury.

More studies are needed, especially in the clinical setting, to assess the immunomodulatory effects of fluoroquinolones.

### Corticosteroids in CAP and the need for new trials

Although the recent RCTs provide data which have increased our knowledge regarding the usefulness of corticosteroids in severe CAP, more studies are needed to clarify the effect of corticosteroids on mortality. Moreover, we need to clarify which corticosteroids and what doses and durations of therapy are most indicated and to define the specific populations that may benefit from this adjunctive treatment, such as severe CAP with a strong inflammatory response or infections with specific pathogens. Another interesting topic is the effect of combination therapy with macrolides and corticosteroids in the modulation of the immune response. We have some promising data from experimental models but more data are needed.

### Inmunoglobulin as adjuntive therapy in CAP

In patients with sepsis and septic shock, low levels of immunoglobulins (Igs) were detected, with a reduction in IgG of between 25 and 61 % and a reduction in IgM of between 19 and 33 % [52]. However, a recent meta-analysis pointed out the limitations of the studies on this topic due to the use of heterogeneous cutoffs to define normal levels of IgG [53].

Hypogammaglobulinemia and low levels of IgG subclasses were noticed in patients with recurrent episodes of pneumonia and may be responsible for the predisposition to recurrent infections [9].

A case–control study [54] showed that patients with CAP had significantly lower levels of IgG (especially IgG2 subclass) and IgA on diagnosis in comparison with a control group of healthy patients without pneumonia. Low levels of Igs persisted in the convalescent phase in approximately 25 % of patients. Hypogammaglobulinemia was more frequently found in patients requiring hospitalization than in outpatients and in patients with pneumonia due to a bacterium other than *Streptococcus pneumoniae* or a virus or without pathogen isolation.

Another study [55] confirmed that severe viral infection due to H1N1 was associated with lower levels of the IgG2 subclass. Indeed, lower levels of Igs appeared to be associated with more severe disease, viral infection, or bacterial infection other than *Streptococcus pneumoniae*.

The reason why patients with pneumonia and sepsis can have low levels of Igs is still not clear. Two different mechanisms may be involved: the infection may be responsible for hypogammaglobulinemia by consuming the Igs or the presence of hypogammaglobulinemia may be the cause of the infection because it contributes to an inadequate defense response. For these reasons, in recent years, it has been suggested that the administration of intravenous Igs may be an effective adjunctive treatment to modulate the immune response in these patients.

Some trials have evaluated the effects of exogenous Igs as adjunctive treatment in patients with sepsis and, in particular, in patients with CAP. However, the results of these studies are still the subject of debate [56].

A meta-analysis [57] reported a general reduction in mortality (approximately 21 %) in adult patients with sepsis and septic shock who received polyclonal Igs and a more evident effect on mortality in the subgroup receiving IgM-enriched immunoglobulin. A more recent meta-analysis by Cochrane published in 2013 [56] showed a reduction in mortality in patients who received polyclonal intravenous Igs, although this positive effect disappeared on analyzing only trials with low bias. A large retrospective study in Japan [58] evaluated the effect of intravenous immunoglobulin as an adjunctive treatment in patients with septic shock due to pneumonia. A total of 8264 patients were studied, of whom 1324 were treated with intravenous Igs, in comparison with 6940 patients who did not receive the Igs. No benefit was found in terms of mortality in the group of patients receiving the Igs.

A multicentre, randomized, placebo-controlled phase II trial [59] is ongoing with the aim of evaluating the efficacy and safety of IgM-enriched immunoglobulin preparations (pentaglobin™, 12 % IgM, 12 % IgA, and 76 % IgG) as adjunctive treatment in patients with mechanical ventilation for CAP. The primary outcome is the number of ventilator-free days.

## Conclusions

The use of corticosteroids in patients with severe CAP and a strong inflammatory reaction can reduce the time to clinical stability and the risk of treatment failure, especially radiological progression. The administration of intravenous immunoglobulins can reinforce the immune response to infection, particularly in patients with inadequate levels of antibodies and when an enriched IgM preparation is used. However, more studies are needed to evaluate their effects in patients with CAP.

## Abbreviations

ARDS, acute respiratory distress syndrome; CAP, community acquired pneumonia; CI, confidence interval; COPD, chronic obstructive pulmonary disease; CRP, C-reactive protein; CSF, colony-stimulating factor; ICU, intensive care unit; Ig, immunoglobulin; IL, interleukin; OR, odds ratio; RCT, randomized controlled trial; RR, risk ratio; TNF, tumor necrosis factor

## References

[1] Shindo Y, Ito R, Kobayashi D, Ando M, Ichikawa M, Goto Y et al. Risk factors for 30-day mortality in patients with pneumonia who receive appropriate initial antibiotics: an observational cohort study. Lancet Infect Dis. 2015;15(9):1055–65. 10.1016/S1473-3099(15)00151-6 26145194

[2] Mongardon N, Max A, Bougle A, Pene F, Lemiale V, Charpentier J (2012). Epidemiology and outcome of severe pneumococcal pneumonia admitted to intensive care unit: a multicenter study. Crit Care.

[3] Annane D, Bellissant E, Cavaillon JM (2005). Septic shock. Lancet.

[4] Antunes G, Evans SA, Lordan JL, Frew AJ (2002). Systemic cytokine levels in community-acquired pneumonia and their association with disease severity. Eur Respir J.

[5] Menendez R, Torres A, Zalacain R, Aspa J, Martin Villasclaras JJ, Borderias L (2004). Risk factors of treatment failure in community acquired pneumonia: implications for disease outcome. Thorax.

[6] Menendez R, Cavalcanti M, Reyes S, Mensa J, Martinez R, Marcos MA (2008). Markers of treatment failure in hospitalised community acquired pneumonia. Thorax.

[7] Darton T, Guiver M, Naylor S, Jack DL, Kaczmarski EB, Borrow R (2009). Severity of meningococcal disease associated with genomic bacterial load. Clin Infect Dis.

[8] Wunderink RG (2015). Corticosteroids for severe community-acquired pneumonia: not for everyone. JAMA.

[9] Notarangelo LD, Fischer A, Geha RS, Casanova JL, Chapel H, Conley ME (2009). Primary immunodeficiencies: 2009 update. J Allergy Clin Immunol.

[10] Rhen T, Cidlowski JA (2005). Antiinflammatory action of glucocorticoids--new mechanisms for old drugs. N Engl J Med.

[11] Meijvis SC, van de Garde EM, Rijkers GT, Bos WJ (2012). Treatment with anti-inflammatory drugs in community-acquired pneumonia. J Intern Med.

[12] Maxime V, Lesur O, Annane D (2009). Adrenal insufficiency in septic shock. Clin Chest Med.

[13] Sibila O, Luna CM, Agusti C, Baquero S, Gando S, Patron JR (2008). Effects of glucocorticoids in ventilated piglets with severe pneumonia. Eur Respir J.

[14] Meduri GU, Kanangat S, Bronze M, Patterson DR, Meduri CU, Pak C (2001). Effects of methylprednisolone on intracellular bacterial growth. Clin Diagn Lab Immunol.

[15] Confalonieri M, Urbino R, Potena A, Piattella M, Parigi P, Puccio G (2005). Hydrocortisone infusion for severe community-acquired pneumonia: a preliminary randomized study. Am J Respir Crit Care Med.

[16] Garcia-Vidal C, Calbo E, Pascual V, Ferrer C, Quintana S, Garau J (2007). Effects of systemic steroids in patients with severe community-acquired pneumonia. Eur Respir J.

[17] Snijders D, Daniels JM, de Graaff CS, van der Werf TS, Boersma WG (2010). Efficacy of corticosteroids in community-acquired pneumonia: a randomized double-blinded clinical trial. Am J Respir Crit Care Med.

[18] Meijvis SC, Hardeman H, Remmelts HH, Heijligenberg R, Rijkers GT, van Velzen-Blad H (2011). Dexamethasone and length of hospital stay in patients with community-acquired pneumonia: a randomised, double-blind, placebo-controlled trial. Lancet.

[19] Chen Y, Li K, Pu H, Wu T. Corticosteroids for pneumonia. Cochrane Database Syst Rev. 2011;(3):CD007720. 10.1002/14651858.CD007720.pub2 21412908

[20] Nie W, Zhang Y, Cheng J, Xiu Q (2012). Corticosteroids in the treatment of community-acquired pneumonia in adults: a meta-analysis. PLoS One.

[21] Shafiq M, Mansoor MS, Khan AA, Sohail MR, Murad MH (2013). Adjuvant steroid therapy in community-acquired pneumonia: a systematic review and meta-analysis. J Hosp Med.

[22] Cheng M, Pan ZY, Yang J, Gao YD (2014). Corticosteroid therapy for severe community-acquired pneumonia: a meta-analysis. Respir Care.

[23] Torres A, Sibila O, Ferrer M, Polverino E, Menendez R, Mensa J (2015). Effect of corticosteroids on treatment failure among hospitalized patients with severe community-acquired pneumonia and high inflammatory response: a randomized clinical trial. JAMA.

[24] Blum CA, Nigro N, Briel M, Schuetz P, Ullmer E, Suter-Widmer I (2015). Adjunct prednisone therapy for patients with community-acquired pneumonia: a multicentre, double-blind, randomised, placebo-controlled trial. Lancet.

[25] Siemieniuk RA, Meade MO, onso-Coello P, Briel M, Evaniew N, Prasad M et al. Corticosteroid therapy for patients hospitalized with community-acquired pneumonia: a systematic review and meta-analysis. Ann Intern Med. 2015. doi:10.7326/M15-0715. [Epub ahead of print]. 10.7326/M15-0715 26258555

[26] Wan YD, Sun TW, Liu ZQ, Zhang SG, Wang LX, Kan QC (2016). Efficacy and safety of corticosteroids for community-acquired pneumonia: a systematic review and meta-analysis. Chest.

[27] Fine MJ, Auble TE, Yealy DM, Hanusa BH, Weissfeld LA, Singer DE (1997). A prediction rule to identify low-risk patients with community-acquired pneumonia. N Engl J Med.

[28] Briel M, Boscacci R, Furrer H, Bucher HC (2005). Adjunctive corticosteroids for Pneumocystis jiroveci pneumonia in patients with HIV infection: a meta-analysis of randomised controlled trials. BMC Infect Dis.

[29] Cao B, Gao H, Zhou B, Deng X, Hu C, Deng C et al. Adjuvant corticosteroid treatment in adults with influenza A (H7N9) viral pneumonia. Crit Care Med. 2016;44(6):e318-328. 10.1097/CCM.0000000000001616 26934144

[30] Rodrigo C, Leonardi-Bee J, Nguyen-Van-Tam J, Lim WS (2016). Corticosteroids as adjunctive therapy in the treatment of influenza. Cochrane Database Syst Rev.

[31] Delaney JW, Pinto R, Long J, Lamontagne F, Adhikari NK, Kumar A (2016). The influence of corticosteroid treatment on the outcome of influenza A(H1N1pdm09)-related critical illness. Crit Care.

[32] Ranzani OT, Ferrer M, Esperatti M, Giunta V, Bassi GL, Carvalho CR (2012). Association between systemic corticosteroids and outcomes of intensive care unit-acquired pneumonia. Crit Care Med.

[33] Donihi AC, Raval D, Saul M, Korytkowski MT, DeVita MA (2006). Prevalence and predictors of corticosteroid-related hyperglycemia in hospitalized patients. Endocr Pract.

[34] Baker EH, Janaway CH, Philips BJ, Brennan AL, Baines DL, Wood DM (2006). Hyperglycaemia is associated with poor outcomes in patients admitted to hospital with acute exacerbations of chronic obstructive pulmonary disease. Thorax.

[35] Chakrabarti B, Angus RM, Agarwal S, Lane S, Calverley PM (2009). Hyperglycaemia as a predictor of outcome during non-invasive ventilation in decompensated COPD. Thorax.

[36] van den BG, Wouters P, Weekers F, Verwaest C, Bruyninckx F, Schetz M (2001). Intensive insulin therapy in the critically ill patients. N Engl J Med.

[37] van den BG, Wilmer A, Hermans G, Meersseman W, Wouters PJ, Milants I (2006). Intensive insulin therapy in the medical ICU. N Engl J Med.

[38] Pereira RM, de Freire CJ (2011). Glucocorticoid-induced myopathy. Joint Bone Spine.

[39] De JB, Sharshar T, Lefaucheur JP, Authier FJ, Durand-Zaleski I, Boussarsar M (2002). Paresis acquired in the intensive care unit: a prospective multicenter study. JAMA.

[40] Mandell LA, Wunderink RG, Anzueto A, Bartlett JG, Campbell GD, Dean NC (2007). Infectious Diseases Society of America/American Thoracic Society consensus guidelines on the management of community-acquired pneumonia in adults. Clin Infect Dis.

[41] Amsden GW (2005). Anti-inflammatory effects of macrolides--an underappreciated benefit in the treatment of community-acquired respiratory tract infections and chronic inflammatory pulmonary conditions?. J Antimicrob Chemother.

[42] Kovaleva A, Remmelts HH, Rijkers GT, Hoepelman AI, Biesma DH, Oosterheert JJ (2012). Immunomodulatory effects of macrolides during community-acquired pneumonia: a literature review. J Antimicrob Chemother.

[43] Postma DF, van Werkhoven CH, van Elden LJ, Thijsen SF, Hoepelman AI, Kluytmans JA (2015). Antibiotic treatment strategies for community-acquired pneumonia in adults. N Engl J Med.

[44] Garin N, Genne D, Carballo S, Chuard C, Eich G, Hugli O (2014). beta-Lactam monotherapy vs beta-lactam-macrolide combination treatment in moderately severe community-acquired pneumonia: a randomized noninferiority trial. JAMA Intern Med.

[45] Tagliabue C, Techasaensiri C, Torres JP, Katz K, Meek C, Kannan TR (2011). Efficacy of increasing dosages of clarithromycin for treatment of experimental Mycoplasma pneumoniae pneumonia. J Antimicrob Chemother.

[46] Lorenzo MJ, Moret I, Sarria B, Cases E, Cortijo J, Mendez R (2015). Lung inflammatory pattern and antibiotic treatment in pneumonia. Respir Res.

[47] Polverino E, Cilloniz C, Dambrava P, Gabarrus A, Ferrer M, Agusti C (2013). Systemic corticosteroids for community-acquired pneumonia: reasons for use and lack of benefit on outcome. Respirology.

[48] Crisafulli E, Menéndez R, Huerta A, Martinez R, Montull B, Clini E et al. Systemic inflammatory pattern of community-acquired pneumonia (CAP) patients with and without chronic obstructive pulmonary disease (COPD). Chest. 2013;143(4):1009–17. 10.1378/chest.12-1684 23187314

[49] Ferrer M, Torres A, Martinez R, Ramirez P, Polverino E, Montull B (2014). Inhaled corticosteroids and systemic inflammatory response in community-acquired pneumonia: a prospective clinical study. Respirology.

[50] Huerta A, Crisafulli E, Menendez R, Martinez R, Soler N, Guerrero M et al. Pneumonic and non-pneumonic exacerbations of COPD: systemic inflammatory response and clinical characteristics. Chest. 2013. doi:10.1378/chest.13-0488. [Epub ahead of print]. 10.1378/chest.13-0488 23828375

[51] Dalhoff A, Shalit I (2003). Immunomodulatory effects of quinolones. Lancet Infect Dis.

[52] Venet F, Gebeile R, Bancel J, Guignant C, Poitevin-Later F, Malcus C (2011). Assessment of plasmatic immunoglobulin G, A and M levels in septic shock patients. Int Immunopharmacol.

[53] Shankar-Hari M, Culshaw N, Post B, Tamayo E, Andaluz-Ojeda D, Bermejo-Martin JF (2015). Endogenous IgG hypogammaglobulinaemia in critically ill adults with sepsis: systematic review and meta-analysis. Intensive Care Med.

[54] de la Torre MC, Bolibar I, Vendrell M, De GJ, Vendrell E, Rodrigo MJ (2013). Serum immunoglobulins in the infected and convalescent phases in community-acquired pneumonia. Respir Med.

[55] Gordon CL, Langan K, Charles PG, Bellomo R, Hart GK, Torresi J (2011). Pooled human immunoglobulin therapy in critically Ill patients with pandemic 2009 influenza A(H1N1) pneumonitis and immunoglobulin G2 subclass (IgG2) deficiency. Clin Infect Dis.

[56] Alejandria MM, Lansang MA, Dans LF, Mantaring JB (2013). Intravenous immunoglobulin for treating sepsis, severe sepsis and septic shock. Cochrane Database Syst Rev.

[57] Kreymann KG, De HG, Nierhaus A, Kluge S (2007). Use of polyclonal immunoglobulins as adjunctive therapy for sepsis or septic shock. Crit Care Med.

[58] Tagami T, Matsui H, Fushimi K, Yasunaga H (2015). Intravenous immunoglobulin and mortality in pneumonia patients with septic shock: an observational nationwide study. Clin Infect Dis.

[59] Welte T, Dellinger RP, Ebelt H, Ferrer M, Opal SM, Schliephake DE (2015). Concept for a study design in patients with severe community-acquired pneumonia: a randomised controlled trial with a novel IGM-enriched immunoglobulin preparation--The CIGMA study. Respir Med.

